# Retrospective comparison of ChatGPT-4 treatment recommendations with real-world physician management in newly diagnosed hypertension: a single-centre study

**DOI:** 10.3389/fcvm.2026.1808572

**Published:** 2026-05-12

**Authors:** Kadri Murat Gürses, Hüseyin Tezcan, Abdullah Tunçez, Yasin Özen, Muhammed Ulvi Yalçın

**Affiliations:** School of Medicine, Selcuk University, Konya, Türkiye

**Keywords:** antihypertensive therapy, artificial intelligence, blood pressure control, ChatGPT-4, clinical decision-making, hypertension, large language models, treatment concordance

## Abstract

**Background:**

Despite the availability of effective first-line therapies, hypertension remains underdiagnosed and undertreated worldwide. Large language models (LLMs), such as ChatGPT-4, may support guideline-adherent prescribing, yet their role in chronic disease management has not been adequately studied.

**Objectives:**

To assess the concordance between ChatGPT-4’s antihypertensive treatment recommendations and real-world physician prescriptions, and to evaluate the association between treatment concordance and short-term blood pressure (BP) control.

**Methods:**

This retrospective, single-centre study included 100 newly diagnosed, treatment-naïve hypertensive adults aged 18–65 years. For each patient, anonymized clinical data were retrospectively entered into ChatGPT-4 after the clinical encounter using a standardized prompt based on ESC/ESH and ACC/AHA guidelines. These recommendations were generated retrospectively after the clinical encounter and were not available to the treating physicians, who were therefore unaware of the ChatGPT-4 outputs.ChatGPT-4 recommendations were generated for study comparison only and did not influence physician prescribing or patient management. The primary outcome was concordance between AI and physician treatment decisions. Secondary outcomes included BP control at 30 ± 5 days and the distribution of therapy classes among uncontrolled cases. Statistical analyses included Cohen’s *κ*, *χ*² testing, and multivariable logistic regression.

**Results:**

ChatGPT-4 and physician treatment choices showed substantial agreement (κ = 0.67). Guideline-recommended dual therapy (e.g., ACEi + thiazide) was selected in 74% of ChatGPT-4 and 67% of physician cases. BP control was achieved in 73% of concordant cases versus 40% of discordant cases. Concordance was independently associated with BP control (adjusted OR = 3.8; 95% CI: 1.5–9.5). Among uncontrolled cases, ChatGPT-4 more frequently recommended fixed-dose combination therapy.

**Conclusion:**

ChatGPT-4 demonstrated strong concordance with expert prescribing patterns, and concordant cases had higher rates of early BP control. Because ChatGPT-4 recommendations were generated retrospectively and did not guide treatment, these findings should be interpreted as showing an association rather than causation, and they warrant prospective validation.

## Introduction

Hypertension (HTN) is often referred to as the “silent killer” because it progresses asymptomatically while gradually damaging the heart, brain, kidneys, and vasculature. Worldwide, more than one billion adults are affected; however, fewer than half are diagnosed, and only approximately one in five achieves adequate blood pressure (BP) control—contributing to an estimated 10.8 million deaths annually (1). Across all levels of care, early recognition and timely, evidence-based treatment are essential to prevent outcomes such as stroke, myocardial infarction, and renal failure (2).

Artificial intelligence (AI) is increasingly being incorporated into clinical decision-making, particularly in acute care settings. Applications such as emergency department triage, automated detection of myocardial infarction on electrocardiograms, and early prediction of clinical deterioration have shown gains in diagnostic accuracy and workflow efficiency (3–7). However, most existing evaluations have focused on time-critical, high-acuity conditions. Within hypertension specifically, most published AI applications have focused on BP measurement, diagnosis, prognostication, or digital monitoring rather than direct pharmacologic decision support (8–11).

Hypertension is a particularly suitable disease model for evaluating AI-supported prescribing because it is common, longitudinal, and managed using structured guideline-based pathways. Initial treatment decisions are typically based on standardized variables routinely available in practice—such as repeated BP values, comorbidities, renal function, electrolytes, and overall cardiovascular risk—making newly diagnosed HTN a pragmatic setting in which to test whether an LLM can translate structured clinical inputs into guideline-consistent first-line treatment recommendations (10, 12). This population is also clinically heterogeneous at presentation, with variation in circadian BP pattern, vascular stiffness, and responses to lifestyle exposures, reinforcing the relevance of individualized early management (13, 14).

In this study, we examined whether newly diagnosed hypertension, a common chronic disease with clear guideline-based treatment strategies, provides a useful setting to assess the practical utility of large language models (LLMs) in routine prescribing. To address the limited evidence on LLM use in hypertension management, we retrospectively compared ChatGPT-4 treatment recommendations with real-world cardiologist decisions and one-month BP outcomes in 100 incident HTN cases at our center. Our objective was to evaluate treatment concordance, BP control, and alignment with contemporary guidelines, thereby clarifying both the potential and the current limitations of LLM-assisted decision support in routine hypertension management.

## Methods

### Study design and setting

This single-centre, retrospective observational study was conducted at tertiary care hospital in Konya, Türkiye. The study was performed in accordance with the principles of the Declaration of Helsinki and received approval from the Necmettin Erbakan University Ethics Committee (approval number: 2024/5285).

Data were accessed for research purposes between 20 October 2024 and 15 November 2024 from the electronic health records of of the study centre. All datasets were fully anonymized prior to analysis, and the authors did not have access to any directly identifiable personal information during or after data collection.

### Participants

Between 15 October 2022 and 15 October 2024, electronic health records were screened for adults aged 18–65 years who had been newly diagnosed with essential hypertension and had no prior history of antihypertensive medication use. Patients were included if they had a complete baseline dataset and attended their scheduled follow-up visit at the dedicated blood pressure clinic within 30 ± 5 days.

Exclusion criteria included: use of any antihypertensive agent for any indication prior to diagnosis, stage 4 or higher chronic kidney disease (eGFR < 30 mL·min^−^¹·1.73 m^2^), decompensated heart failure, chronic liver disease, any identifiable secondary cause of hypertension, or missing outcome data.

### Data collection

At the index visit, a comprehensive clinical summary was generated for each participant. This included demographic information, comorbid conditions, three consecutive seated blood pressure measurements, routine biochemical parameters (urea, creatinine, sodium, potassium, and calculated eGFR), and a complete lipid profile (LDL-C, HDL-C, and triglycerides). Office blood pressure was measured by trained clinic staff using a validated [automated oscillometric device/calibrated manual sphygmomanometer] with an appropriately sized cuff after at least 5 min of seated rest. Three consecutive readings were obtained at 1–2 min intervals, and the [mean of the last two readings/average of all three readings] was used for analysis. Hematological indices, including hemoglobin, white blood cell count, and platelet count, were also recorded. All laboratory analyses were performed in the hospital’s ISO 15189-accredited core laboratory, operating under both internal and external quality assurance protocols. The anonymized case records were exported as comma-separated value (CSV) files and used without modification as input for ChatGPT-4. An illustrative example of the structured case input and the standardized prompt used for model querying is provided in Supplementary File S1.

### ChatGPT-4 protocol

For each patient, the full anonymised case summary (demographics, vitals, laboratory results and comorbidities) was pasted into ChatGPT-4 (OpenAI, model gpt-4-turbo-preview; temperature 0.2) with the prompt:

“You are a cardiology specialist. Recommend **first-line pharmacotherapy** for this newly diagnosed, treatment-naïve hypertensive adult. Choose from: ACE-inhibitor, ARB, thiazide diuretic, β-blocker, dihydropyridine- or non-dihydropyridine calcium-channel blocker (CCB), spironolactone. You may recommend monotherapy or a two-drug fixed-dose combination in line with contemporary major guidelines (ESC/ESH 2023, ACC/AHA 2017)”.

ChatGPT-4 recommendations were generated retrospectively from anonymized case summaries after the original prescribing decision had already been made and were not disclosed to the treating cardiologists; therefore, treating physicians were unaware of the model outputs during clinical management.

The model’s recommendation was recorded verbatim and mapped to predefined treatment categories.

The model’s recommendation was recorded verbatim and mapped to one of the predefined antihypertensive regimen categories shown in Table 2. Physician prescriptions were categorized using the same class-based framework. Concordance was defined as exact agreement between the ChatGPT-4 recommendation and the physician’s initial prescription at the regimen-category level. Thus, recommendations were considered concordant only if both fell into the same predefined category; similar but non-identical regimens (for example, ACEi + thiazide vs ARB + thiazide, or ACEi monotherapy vs ARB monotherapy) were classified as discordant. Because the comparison was performed at the drug-class/regimen level, agent-level differences within the same class were not analyzed separately.

### Outcomes

*Primary outcome* – concordance between the ChatGPT recommendation and the cardiologist’s initial prescription, assessed as categorical agreement.

**Figure 1:**
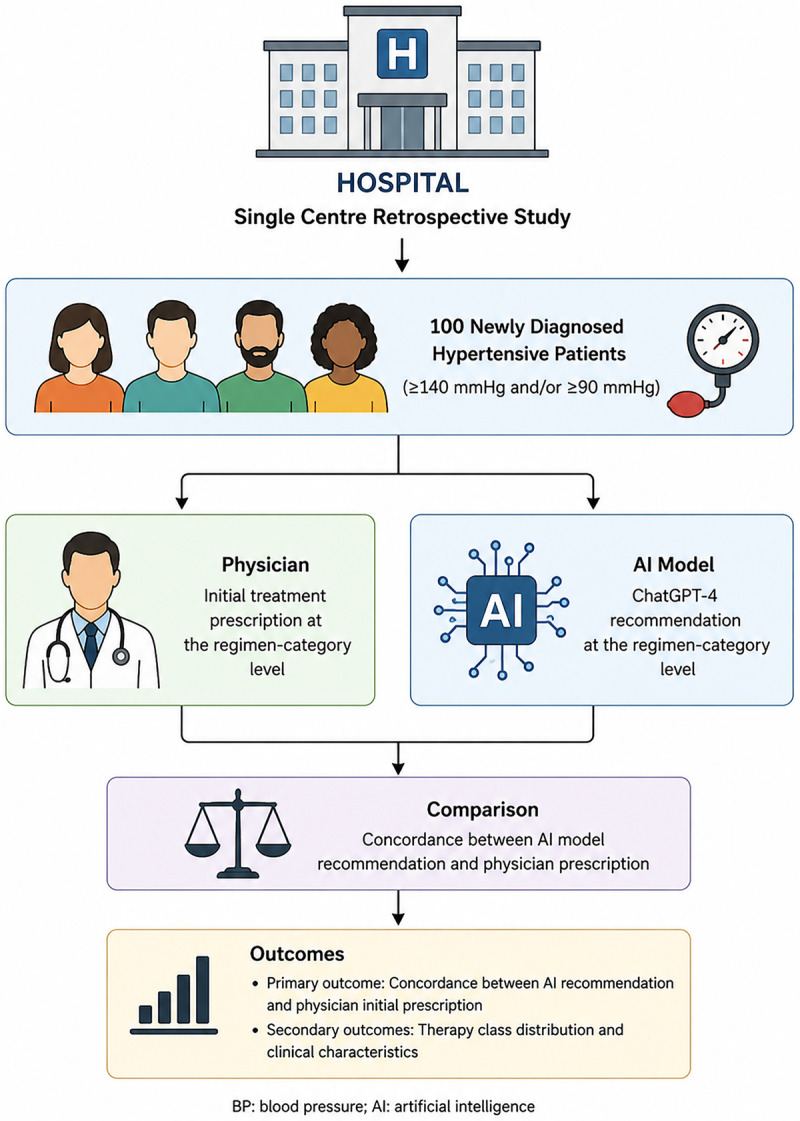
Study flowchart: AI vs physician treatment comparison in hypertension. Visual summary of the study design and key endpoints. One hundred newly diagnosed hypertensive patients (BP ≥140/90 mmHg) at tertiary care hospital in Konya were retrospectively analyzed. Initial treatment recommendations from ChatGPT-4 were compared to those of physicians. Primary outcome was treatment concordance; secondary outcomes included blood pressure control at one month and analysis of therapy class distributions in uncontrolled cases.

*Secondary outcomes* – (i) proportion of patients achieving BP < 140/90 mmHg at one-month follow-up; (ii) distribution of therapy classes among uncontrolled cases, comparing ChatGPT suggestions with physician management. A visual summary of the study design, patient selection, and outcome measures is provided in Figure 1.

### Statistical analysis

Treatment categories were cross-tabulated, and agreement between ChatGPT-4 recommendations and physician prescriptions was quantified using Cohen’s *κ* statistic with 95 % confidence intervals (CI). Continuous variables are presented as mean ± standard deviation (SD) and compared using Student’s *t*-test. Categorical variables are expressed as counts and percentages [*n* (%)] and compared using the *χ*² test or Fisher’s exact test, as appropriate.

To identify independent predictors of blood pressure control at one month, multivariable logistic regression was performed, adjusting for age, sex, baseline systolic BP, and diabetes status. A two-sided *p*-value of <0.05 was considered statistically significant. All analyses were conducted using R software, version 4.3. Because the cohort size was modest and the number of uncontrolled follow-up outcomes was limited, the multivariable model was intentionally kept parsimonious to reduce the risk of overfitting and unstable estimates. Covariates were selected *a priori* based on clinical relevance and potential confounding of short-term blood pressure control.

## Results

### Baseline characteristics

A total of 100 treatment-naïve adults with newly diagnosed essential hypertension met the inclusion criteria (Table 1). The cohort was middle-aged (mean age 51 ± 9 years) and predominantly male (58%). The mean baseline blood pressure was 154 ± 11/96 ± 7 mmHg, and renal function was generally preserved (mean eGFR 82 ± 15 mL·min^−^^1^·1.73 m^2^). Diabetes mellitus was present in 22% of participants, and 27% were current smokers. Established cardiovascular disease (CVD) was relatively uncommon, occurring in 8% of the cohort.

**Table 1:** Baseline characteristics of the study population (*n* = 100).

Variable	Value ± SD or *n* (%)
Age, yr	51 ± 9
Male sex	58 (58 %)
BMI, kg·m^−^²	29.1 ± 4.2
Current smoker	27 (27 %)
Diabetes mellitus	22 (22 %)
Pre-existing CVD	8 (8 %)
Systolic BP, mmHg	154 ± 11
Diastolic BP, mmHg	96 ± 7
Urea, mg·dL^−^^1^	34 ± 8
Creatinine, mg·dL^−^^1^	0.9 ± 0.2
eGFR, mL·min^−^^1^·1.73 m^−^^2^	82 ± 15
Na^+^, mmol·L^−^^1^	139 ± 3
K^+^, mmol·L^−^^1^	4.2 ± 0.4
Haemoglobin, g·dL^−^^1^	14.3 ± 1.4
WBC, 10^3^·µL^−^^1^	7.2 ± 1.8
Platelets, 10^3^·µL^−^^1^	248 ± 56
LDL-C, mg·dL^−^^1^	132 ± 27
HDL-C, mg·dL^−^^1^	46 ± 9
Triglycerides, mg·dL^−^^1^	158 ± 62

BMI, body mass index; BP, blood pressure; CVD, cardiovascular disease; eGFR, estimated glomerular filtration rate; Na^+^, sodium; K^+^, potassium; WBC, white blood cell count; LDL C, low-density lipoprotein cholesterol; HDL C, high-density lipoprotein cholesterol.

### Initial antihypertensive therapy selections

Both ChatGPT-4 and the treating physicians predominantly recommended dual therapy regimens combining a renin–angiotensin system inhibitor—either an angiotensin-converting enzyme inhibitor (ACEi) or an angiotensin receptor blocker (ARB)—with either a thiazide diuretic or a dihydropyridine calcium channel blocker (CCB). These four combinations (ACEi + thiazide, ACEi + CCB, ARB + thiazide, and ARB + CCB) are consistently endorsed as preferred initial treatments in major hypertension guidelines.

Physicians prescribed one of these guideline-recommended combinations in 67% of cases, most frequently ACEi + thiazide (30%). ChatGPT-4 recommended one of the four preferred combinations in 74% of cases, also most commonly ACEi + thiazide (34%). Full details of treatment distributions are provided in Table 2. Overall agreement between human and AI selections was 80%, corresponding to a Cohen’s κ of 0.67 (95% CI: 0.55–0.79), indicating substantial concordance.

**Table 2:** Initial antihypertensive therapy recommendations.

Treatment regimen	Physician *n* (%)	ChatGPT-4 *n* (%)
ACE I ± Thiazide	30 (30%)	34 (34%)
ARB ± Thiazide	12 (12%)	14 (14%)
ACE I ± CCB	15 (15%)	14 (14%)
ARB ± CCB	10 (10%)	12 (12%)
ACE I monotherapy	8 (8%)	7 (7%)
ARB monotherapy	6 (6%)	5 (5%)
CCB monotherapy	5 (5%)	6 (6%)
BB monotherapy	3 (3%)	2 (2%)
ACE I ± BB	3 (3%)	2 (2%)
ARB ± BB	2 (2%)	1 (1%)
BB ± Thiazide	2 (2%)	1 (1%)
BB ± CCB	2 (2%)	1 (1%)
CCB ± Thiazide	2 (2%)	1 (1%)
Total	100 (100%)	100 (100%)

Overall agreement between physician and ChatGPT-4 treatment selections was 80%, with substantial concordance (Cohen’s κ = 0.67; 95% CI: 0.55–0.79).

ACEi, angiotensin-converting enzyme inhibitor; ARB, angiotensin receptor blocker; BB, β-blocker; CCB, calcium channel blocker.

### Blood-pressure control at 30 ± 5 days

**Figure 2:**
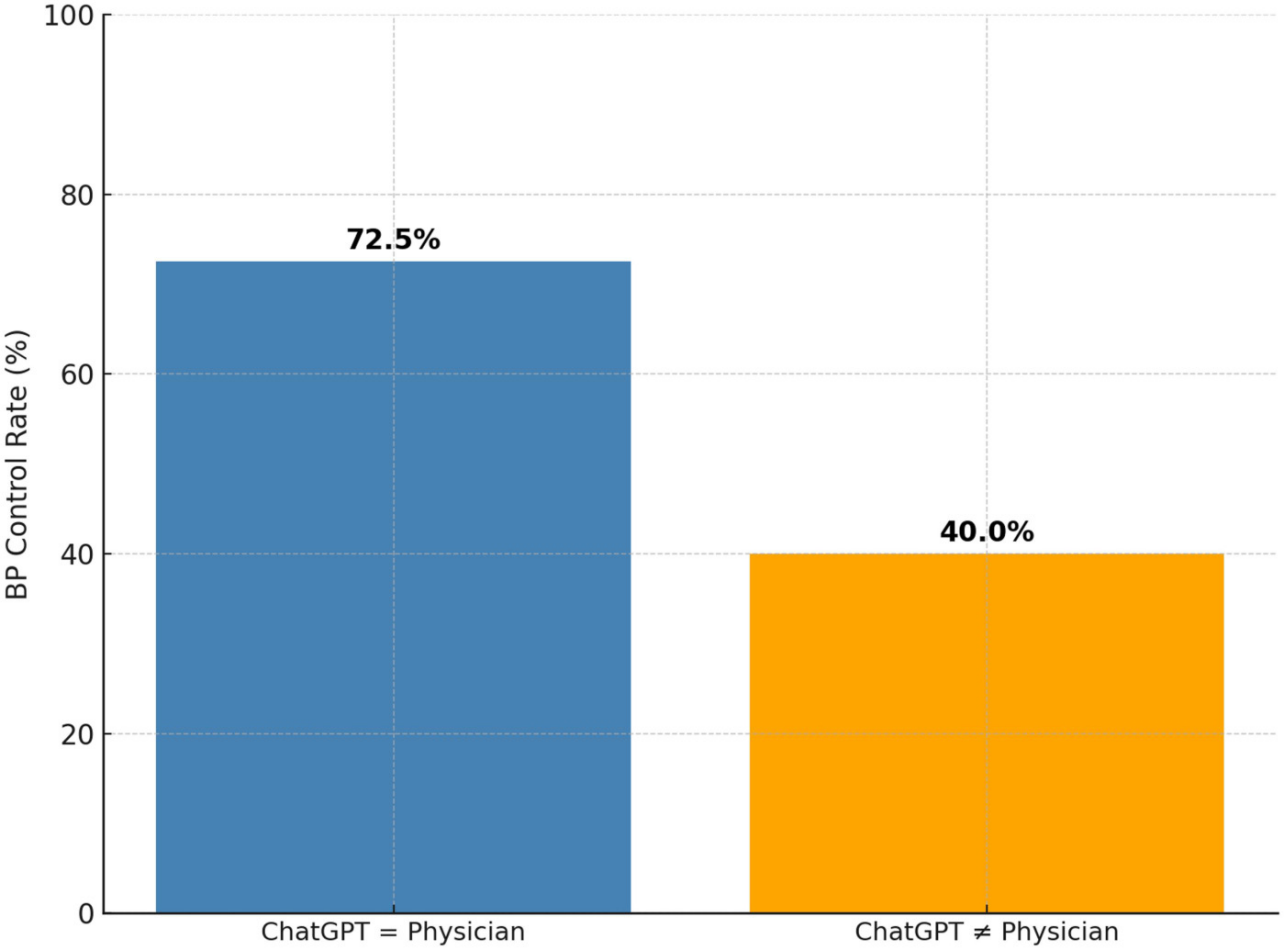
Impact of treatment concordance on blood pressure control. Comparison of blood pressure control rates at one-month follow-up between patients whose initial antihypertensive therapy was concordant between ChatGPT-4 and the treating physician versus those whose treatment differed BP control (<140/90 mmHg) was achieved in 72% of concordant cases compared with 40% of discordant cases.

At follow-up, 66 patients (66%) achieved blood pressure control, defined as <140/90 mmHg. Control rates varied substantially based on concordance between the physician’s prescription and ChatGPT-4’s recommendation (Table 3). Among the 80 patients for whom the prescribed treatment matched the model’s suggestion, 58 (72.5%) achieved target BP. In contrast, only 8 of the 20 patients (40.0%) in the discordant group attained control (Figure 2).

**Table 3:** Concordance between ChatGPT-4 recommendations and physician prescriptions, and subsequent blood-pressure control.

Concordance status	Patients (*n*)	BP < 140/90 mmHg at 1 Month *n* (%)
ChatGPT = Physician	80	58 (72.5 %)
ChatGPT ≠ Physician	20	8 (40.0 %)
**Total**	**100**	**66 (66 %)**

Concordance independently predicted BP control (adjusted OR: 3.8, 95 % CI: 1.5–9.5).

BP, blood pressure; OR, odds ratio; CI, confidence interval.

The crude odds of achieving BP control were nearly four times higher in the concordant group (OR: 3.95; 95% CI: 1.43–10.97; *p* = 0.008). After adjusting for age, sex, baseline systolic blood pressure, and diabetes status, concordance remained an independent predictor of BP control (adjusted OR: 3.8; 95% CI: 1.5–9.5).

### Therapy patterns among uncontrolled participants

**Figure 3:**
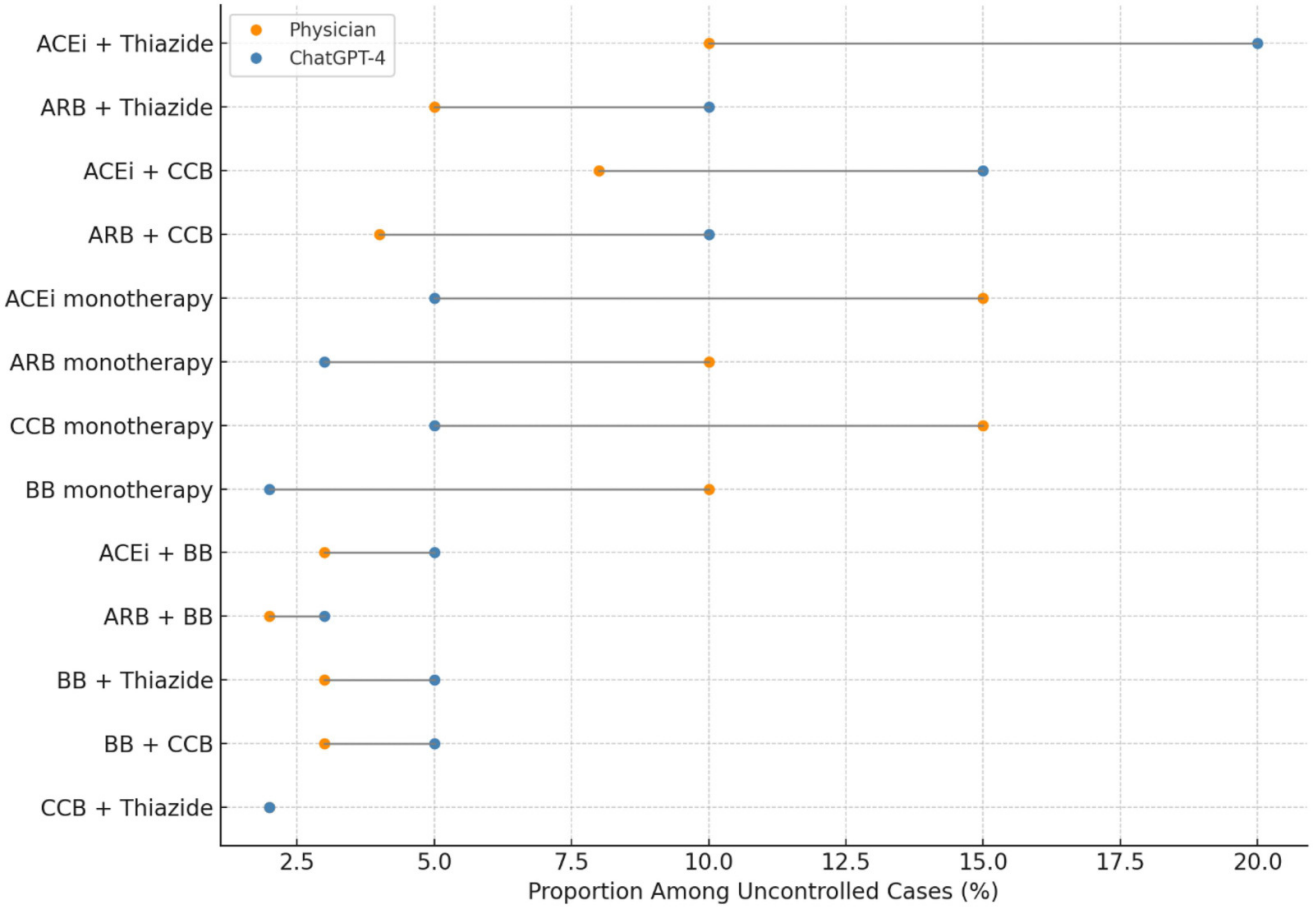
Distribution of individual antihypertensive regimens Among uncontrolled hypertension cases. Lollipop chart comparing the specific antihypertensive regimens used in patients with uncontrolled blood pressure at one-month follow-up. Each line represents one regimen, with dots indicating the proportion of patients for whom the regimen was selected by physicians (orange) or recommended by ChatGPT-4 (blue). ChatGPT-4 more frequently proposed combination therapies such as ACEi + thiazide or ACEi + CCB, whereas physicians tended to favor monotherapy options including ACEi, ARB, or CCB alone. Among uncontrolled cases, ChatGPT-4 more frequently recommended combination regimens, whereas physicians more often selected monotherapy regimens.

Among the 34 patients who remained above target at follow-up, ChatGPT-4 had initially recommended a fixed-dose two-drug combination in 60% of cases, compared with 35% in the physician arm. Detailed regimen distributions among uncontrolled cases are presented in Figure 3.

## Discussion

Despite decades of public health efforts, hypertension continues to pose a formidable global challenge, both in scale and in the consequences of under-treatment. As highlighted in the 2023 WHO global report, the majority of adults with elevated blood pressure remain undiagnosed or undertreated, and millions of preventable deaths occur annually as a result (1). Paradoxically, first-line antihypertensive therapy is both inexpensive and highly effective when initiated promptly. Global modeling estimates suggest that achieving guideline-based blood pressure targets could prevent up to 76 million deaths between 2023 and 2050 (15). These data highlight the urgent need for scalable tools that can reduce diagnostic inertia and promote early, guideline-concordant prescribing at all levels of care (2).

The present findings are best interpreted as a proof-of-concept comparison of treatment recommendations rather than evidence that generative AI improves clinical outcomes in hypertension care (16, 17). In our cohort, ChatGPT-4 showed substantial concordance with cardiologist prescribing, particularly for guideline-preferred initial regimens, which is consistent with broader evidence that newer LLMs can produce clinically plausible recommendations in structured text-based tasks but still show variable accuracy across settings (8, 18). Importantly, because the model outputs were generated retrospectively from anonymized case summaries and were not available to treating physicians, the higher one-month BP control observed in concordant cases should be interpreted as an association within this dataset rather than a treatment effect of AI support (17, 19).

Taken together, recent literature suggests that generative AI is most appropriate in clinical care as a clinician-facing adjunct for structured and reviewable tasks—such as documentation, summarization, information retrieval, medication-safety screening, and preliminary option generation—rather than as an independent prescriber (20–22). This caution is important because LLM outputs can be sensitive to prompt structure and information order, may be non-reproducible or unsafe, and did not significantly improve physician diagnostic reasoning over conventional resources in a randomized trial (16, 21, 23). Accordingly, LLMs should not currently be used autonomously for high-stakes therapeutic decisions, complex multimorbidity, or preference-sensitive scenarios in which patient values, tolerability, feasibility, and clinical uncertainty materially shape the best choice (16, 24). If used in hypertension management, their role at present is better framed as supervised decision support that may help surface guideline-consistent options, while final prescribing remains with the clinician and requires local validation, monitoring, and governance (17–19).

Digital transformation has already embedded artificial intelligence (AI) into acute clinical decision-making. Machine learning–based triage engines now process emergency department queues in real time, effectively identifying high-risk patients and improving clinical throughput metrics, such as door-to-doctor time (25, 26). In cardiology, convolutional neural networks interpret 12-lead electrocardiograms with expert-level accuracy, expediting the diagnosis of ST-elevation myocardial infarction and other life-threatening arrhythmias (27). Similarly, AI-based risk calculators increasingly outperform traditional regression models in cardiovascular risk stratification, enhancing primary prevention strategies (28). These examples highlight AI’s strength in pattern recognition and its reliance on large, annotated datasets—capabilities that have already yielded measurable improvements in diagnostic accuracy and emergency care efficiency.

By contrast, far fewer studies have investigated whether AI can transition from disease recognition to therapeutic decision-making. A recent American Heart Association review noted that most AI applications in hypertension focus on phenotyping or risk prediction, with only a limited number addressing algorithm-guided treatment selection—and even fewer evaluating large language models (LLMs) specifically (29, 30). Early decision-support systems integrated into electronic health records have demonstrated modest improvements in blood pressure control, but these systems rely on rule-based logic and lack the contextual flexibility of modern LLMs (31). Small cluster-randomized trials in India and China have shown that algorithmic reminders can improve adherence to guidelines, yet the algorithms themselves did not generate or recommend specific prescriptions (32, 33). As a result, robust evidence assessing whether generative AI can provide safe and effective antihypertensive treatment recommendations remains limited—an evidence gap that our study aims to address.

We compared ChatGPT-4’s treatment recommendations with the real-world prescribing decisions of board-certified cardiologists in 100 newly diagnosed hypertension cases and observed substantial concordance (κ = 0.67). Notably, patients in whom human and AI recommendations aligned experienced significantly higher rates of blood pressure control at one month. Importantly, ChatGPT-4 recommendations were generated retrospectively from anonymized case summaries after the original clinical encounter and were not available to the treating physicians. Accordingly, these findings should be interpreted as demonstrating concordance and an association with short-term blood pressure control, rather than evidence that AI-supported prescribing improved clinical outcomes. We also observed that, among patients who remained uncontrolled at follow-up, ChatGPT-4 more often recommended fixed-dose combination therapy than was prescribed in routine practice. This pattern may reflect less frequent early treatment intensification in discordant cases; however, given the retrospective design and the potential for residual confounding, this interpretation should be considered hypothesis-generating rather than causal. However, because baseline characteristics were not separately compared between concordant and discordant groups, underlying between-group differences may have contributed to the observed disparity in blood pressure control. A retrospective design was deliberately chosen, as randomizing treatment-naïve patients to AI-generated therapy without prior safety validation would raise important ethical concerns—an issue emphasized in recent guidance on clinical trial design for AI technologies (34). The use of existing patient records enabled us to evaluate model performance in a real-world context without withholding evidence-based treatment. As such, our findings provide an ethically appropriate, proof-of-concept evaluation that can inform the development of prospective studies once adequate governance and oversight structures are established.

The substantial but incomplete concordance observed in our study suggests that ChatGPT-4 was able to approximate cardiologist prescribing patterns in structured hypertension scenarios, but that the discordant cases remain clinically important. This interpretation is consistent with recent hypertension-specific benchmarking showing that GPT-4 performed better than other general-purpose LLMs in common hypertension scenarios, yet still remained below hypertension experts in accuracy, safety, and guideline concordance (18). The higher one-month BP control observed in concordant cases may therefore reflect that concordance identified relatively straightforward, guideline-aligned cases rather than demonstrating a therapeutic effect of AI itself (23, 35). The more frequent recommendation of fixed-dose combination therapy by ChatGPT-4 among patients who remained uncontrolled at follow-up is also noteworthy, because contemporary real-world evidence, and suggests that combination-pill therapy is associated with better medication adherence, fewer hypertension-related emergency visits and hospitalizations, and lower costs than comparable multi-pill therapy (36). Taken together, these findings suggest that LLMs may be most useful in surfacing guideline-consistent initial regimens and prompting consideration of treatment intensity, while the observed outcome differences in this retrospective study may still reflect baseline differences, treatment complexity, or adherence rather than model benefit alone (18, 23, 35, 36).

Alternative explanations for the observed findings should also be considered. Because baseline characteristics were not separately compared between concordant and discordant groups, residual differences in baseline risk profile may have influenced one-month BP control. Blood pressure control is multifactorial and has been linked to patient-level, clinician-level, and health-system factors, including medication adherence and therapeutic inertia (37, 38). Treatment complexity may also have contributed, since fixed-dose combination antihypertensive therapy has been associated with higher rates of BP control than comparable multi-pill regimens in recent real-world analyses (39). Accordingly, the higher control rate observed in concordant cases should be interpreted as an association within this retrospective cohort rather than evidence that concordance itself improved outcomes (37, 40).

One finding that merits further discussion is that, among patients who remained uncontrolled at one month, ChatGPT-4 more often recommended early fixed-dose combination therapy whereas physicians more often prescribed monotherapy. Contemporary hypertension guidance increasingly supports initial two-drug therapy, preferably as a single-pill combination, for most patients who require antihypertensive medication, because this approach can simplify prescribing and accelerate blood pressure reduction (41, 42). Recent real-world data from a large US health system further showed that initial combination therapy was associated with better post-treatment blood pressure control than monotherapy, even though its use declined over time (43). In parallel, recent evidence suggests that combination-pill therapy is associated with higher medication adherence, fewer hypertension-related emergency visits and hospitalizations, and lower costs than multi-pill therapy, while trial-based analyses indicate that low-dose combination strategies may reduce therapeutic inertia compared with stepped monotherapy (36, 44). Accordingly, the greater tendency of ChatGPT-4 to recommend fixed-dose combination therapy may reflect closer alignment with current treatment-intensification strategies. However, because this was a retrospective study with short follow-up and no direct measurement of adherence or treatment escalation after the initial prescription, this finding should be interpreted as exploratory rather than as proof that treatment choice alone drove persistent hypertension in discordant cases.

If future prospective studies confirm that large language model (LLM) recommendations can be integrated safely and effectively into clinical workflows, such tools may have health-economic and implementation value. The substantial but incomplete concordance observed in our study suggests that ChatGPT-4 was able to approximate cardiologist prescribing patterns in structured hypertension scenarios, but that the discordant cases remain clinically important. This interpretation is consistent with recent hypertension-specific benchmarking showing that GPT-4 performed better than other general-purpose LLMs in common hypertension scenarios, yet still remained below hypertension experts in accuracy, safety, and guideline concordance (18). The higher one-month BP control observed in concordant cases may therefore reflect that concordance identified relatively straightforward, guideline-aligned cases rather than demonstrating a therapeutic effect of AI itself (23, 35). The more frequent recommendation of fixed-dose combination therapy by ChatGPT-4 among patients who remained uncontrolled at follow-up is also noteworthy, because contemporary real-world evidence, and suggests that combination-pill therapy is associated with better medication adherence, fewer hypertension-related emergency visits and hospitalizations, and lower costs than comparable multi-pill therapy (36). Taken together, these findings suggest that LLMs may be most useful in surfacing guideline-consistent initial regimens and prompting consideration of treatment intensity, while the observed outcome differences in this retrospective study may still reflect baseline differences, treatment complexity, or adherence rather than model benefit alone (18, 23, 35, 36).

Ethically, the present findings support a clinician-in-the-loop role for generative AI rather than autonomous prescribing. Recent systematic reviews of LLM use in healthcare identify recurring concerns related to fairness, bias, non-maleficence, transparency, privacy, and the generation of plausible but inaccurate content, and emphasize that acceptable human oversight must be defined according to the clinical context and potential for harm (45, 46). WHO has likewise warned that large multimodal and generative models can produce false, biased, or incomplete statements and may promote automation bias if difficult clinical choices are inappropriately delegated to them (47). Complementary implementation frameworks now recommend that healthcare AI be deployed for well-defined tasks, with clear statements of intended use, limitations, and risks, together with meaningful human oversight, patient-facing transparency when appropriate, and ongoing post-implementation monitoring (17, 48). In practical terms, generative AI may be appropriate in hypertension care for structured, reviewable tasks such as summarizing case data, recalling guideline-based options, or generating preliminary regimen suggestions, but it should not currently function as a stand-alone decision-maker in high-stakes prescribing, complex multimorbidity, or preference-sensitive decisions (23, 35, 47). This more cautious framing is consistent with current evidence showing that generative AI remains variable across tasks and does not yet reliably outperform expert clinicians in complex real-world clinical reasoning (18, 23, 35).

The present findings should be interpreted as a proof-of-concept comparison of treatment recommendations rather than evidence of clinical benefit from AI-assisted care. In this retrospective cohort, ChatGPT-4 showed substantial concordance with cardiologist prescribing, particularly for guideline-recommended dual therapy regimens, and concordant cases had higher rates of short-term BP control. Among uncontrolled cases, ChatGPT-4 more frequently recommended fixed-dose combination therapy than was prescribed in routine practice. However, because the model outputs were generated retrospectively and were not available to treating physicians, these findings do not demonstrate that AI-supported prescribing improves outcomes in clinical practice. Instead, they suggest that LLM recommendations can approximate expert guideline-based treatment patterns in newly diagnosed hypertension and justify prospective evaluation in real-world settings.

## Limitations

This study has several limitations. First, its retrospective, single-centre design limits the generalizability of the findings to other healthcare settings and populations. The relatively small sample size (*n* = 100) may also have reduced statistical power, particularly for subgroup and multivariable analyses. Second, ChatGPT-4’s recommendations were generated solely from structured clinical inputs and did not account for physician–patient interactions, which can influence real-world prescribing decisions. In addition, although the regression model adjusted for age, sex, baseline systolic blood pressure, and diabetes status, other potentially relevant covariates such as BMI, smoking status, lipid parameters, and pre-existing cardiovascular disease were not included; therefore, residual confounding cannot be excluded. Third, blood pressure outcomes were assessed at one month only, offering no information on long-term adherence, durability of response, or clinical endpoints. Selection bias is also possible because only patients with a complete baseline dataset who attended the scheduled 30 ± 5-day follow-up visit were eligible for inclusion. Fourth, although several baseline variables such as BMI, smoking status, lipid parameters, and pre-existing cardiovascular disease were recorded, they were not included in the multivariable model in order to preserve model parsimony in this modest sample. Accordingly, residual confounding cannot be excluded. Fifth, baseline characteristics were summarized for the overall cohort but were not separately compared between concordant and discordant groups. Therefore, differences in patient characteristics between these groups may have influenced the observed variation in blood pressure control, and the association between treatment concordance and outcome should be interpreted cautiously.

Additionally, although the model was prompted to follow major international guidelines (ESC/ESH and ACC/AHA), regional practice variations and ongoing updates may affect the applicability of its recommendations. Finally, the analysis did not incorporate patient-level considerations such as medication tolerability, side effects, or treatment preferences—factors that are integral to personalized and shared decision-making in clinical care.

## Conclusion

In this retrospective study of newly diagnosed hypertension cases, ChatGPT-4 showed substantial concordance with cardiologist prescribing practices, particularly for guideline-recommended dual therapy regimens. Concordant cases had higher rates of blood pressure control at one month. However, because ChatGPT-4 recommendations were generated retrospectively from anonymized case summaries and did not influence physician treatment decisions, these findings should be interpreted as demonstrating concordance and an association with short-term BP control rather than proof that AI-supported prescribing improves clinical outcomes. Prospective interventional studies are needed to determine whether LLM-assisted prescribing can safely improve patient outcomes in real-world practice.

These findings suggest that large language models may serve as valuable decision-support tools in everyday hypertension management, helping to reinforce evidence-based prescribing and reduce therapeutic inertia. By aligning closely with established treatment guidelines, AI models like ChatGPT-4 have the potential to improve early treatment outcomes when used in collaboration with human clinicians. Prospective, real-world studies are needed to further assess the safety, scalability, and long-term clinical impact of AI-assisted prescribing across diverse healthcare settings.

## Data Availability

Due to patient privacy and ethical considerations, the data are not publicly available. The datasets can be provided by the corresponding author upon reasonable request.
