# Supplementary material for: Retrospective comparison of ChatGPT-4 treatment recommendations with real-world physician management in newly diagnosed hypertension: a single-centre study
Source: Front Cardiovasc Med. 2026 May 12;13:1808572. doi: 10.3389/fcvm.2026.1808572 (PMC13202720; doi:10.3389/fcvm.2026.1808572)
Supplement: Supplementary file 1 [file Supplementaryfile1.docx]

Supplementary File 1. Illustrative Structured Clinical Input Used for ChatGPT-4 Querying

# 1. Standardized prompt and model settings

**Model:** OpenAI GPT-4

| **Prompt used in the study:** *"You are a cardiology specialist. Recommend first-line pharmacotherapy for this newly diagnosed, treatment-naïve hypertensive adult. Choose from: ACE-inhibitor, ARB, thiazide diuretic, β-blocker, dihydropyridine- or non-dihydropyridine calcium-channel blocker (CCB), spironolactone. You may recommend monotherapy or a two-drug fixed-dose combination in line with contemporary major guidelines (ESC/ESH 2023, ACC/AHA 2017)."* |
| --- |

# 2. Variables included in the structured case input

*Each case summary included the following domains before being pasted into ChatGPT-4. Newly diagnosed and treatment-naïve status were conveyed by the standardized prompt shown above.*

| **Domain** | **Variables included** | **Example units / coding** |
| --- | --- | --- |
| Demographics / risk profile | Case ID, age, sex, body mass index (BMI), current smoking status | years; Male/Female; kg/m²; Yes/No |
| Comorbidities | Diabetes mellitus, pre-existing cardiovascular disease (CVD) | Yes/No |
| Office BP readings | Three consecutive seated systolic and diastolic blood pressure readings | mmHg |
| Renal / electrolytes | Urea, creatinine, estimated glomerular filtration rate (eGFR), sodium, potassium | mg/dL; mL/min/1.73 m²; mmol/L |
| Hematology | Hemoglobin, white blood cell count, platelet count | g/dL; ×10³/µL |
| Lipid profile | LDL-C, HDL-C, triglycerides | mg/dL |

# 3. Illustrative example of a single-case input

| **Variable** | **Illustrative value** |
| --- | --- |
| case_id | EXAMPLE_001 |
| age_years | 53 |
| sex | Male |
| bmi_kg_m2 | 29.8 |
| current_smoker | Yes |
| diabetes_mellitus | No |
| pre_existing_cvd | No |
| sbp_reading_1_mmHg | 158 |
| dbp_reading_1_mmHg | 98 |
| sbp_reading_2_mmHg | 156 |
| dbp_reading_2_mmHg | 96 |
| sbp_reading_3_mmHg | 157 |
| dbp_reading_3_mmHg | 97 |
| urea_mg_dL | 34 |
| creatinine_mg_dL | 0.92 |
| egfr_mL_min_1_73m2 | 88 |
| sodium_mmol_L | 139 |
| potassium_mmol_L | 4.2 |
| hemoglobin_g_dL | 14.7 |
| wbc_x10^3_uL | 7.1 |
| platelets_x10^3_uL | 244 |
| ldl_c_mg_dL | 136 |
| hdl_c_mg_dL | 44 |
| triglycerides_mg_dL | 168 |

# 4. Note on reproducibility

**This supplementary example is intended to clarify:** (i) which clinical variables were provided to the model, (ii) how those variables were structured, and (iii) that the prompt was applied to a retrospective, de-identified case summary rather than being used in real-time clinical care.
